# Book Reviews

**Published:** 1827-02

**Authors:** 


### CRITICAL ANALYSES.

Quæ laudanda forent, et quæ culpanda, vicissim

Illa, prius, cretâ; mox hæc, carbone, *notamus*.—PERSIUS.

*A Treatise on the Diseases of Children; with Directions for the Management of Infants from the Birth.* By the late MICHAEL UNDERWOOD, M.D. *Eighth Edition, revised, with Notes and Observations,* by SAMUEL MERRIMAN, M.D. F.L.S. Corresponding Member of the Imperial and Royal Academy of Sciences at Siena.—8vo. pp. 636. Callow and Wilson, London, 1827.

THE present publication has been long anxiously expected by the profession; for, whatever might have been the merits of Dr. UNDERWOOD's book when it was originally published, it could not, in the present day, be considered a satisfactory treatise on infantile diseases. It contained much that might fairly have been omitted, and touched but lightly upon many important subjects which obviously required a more detailed consideration. There was also a triviality of manner in the composition of it, which has not escaped the observation of the present Editor, and which was more likely to attract the attention of mothers and nurses, who might aspire to take upon themselves the duties of physicians in their own families, than of medical students or practitioners.

Upon more than one occasion, we have expressed our favourable anticipations of the forthcoming edition of Dr. Underwood's book, which was known to be under the superintendance of Dr. MERRIMAN, whose extensive experience upon the subject is as well known as his talent is generally admitted. We imagined, however, that the new edition was to be prepared upon an entirely different plan. We were in hopes that, although, as a mark of respect, the name of the original author might still be allowed to appear in the title-page, each chapter nevertheless would have been remodelled; and that the profession would have had the benefit of the general results of Dr. Merriman's observations, unincumbered by the errors which subsequent experience has detected in the performance of Dr. Underwood. We confess that we are disappointed; and we are much deceived if the regret will not be universal, when it is seen that Dr. Merriman has added but a few short foot-notes, in explanation, or correction of errors which are still allowed to occupy the pages of the book.

In 1819, Dr. Underwood, then far advanced in years, revised the last edition of his work, which is now out of print, and the proprietors applied to the present editor to prepare a new edition for the press. We sincerely wish that he, who possesses the ability of giving an original work to the public upon the same subject, had not undertaken the subordinate office of supervising and correcting the labours of another.

It would appear from the preface, that Dr. Merriman does not consider himself responsible for the doctrines contained throughout the work. "If the difference of opinion does not involve any material point of practice, he has not always thought it necessary to express dissent; but, on other occasions, he has inserted in a note what he considers to be preferable doctrine; and, throughout the volume, the editor has marked with the initials of his name the opinions and remarks for which he is answerable." The notes which have thus been added by Dr. Merriman would occupy, at most, about six pages of the book.

It appears from the preface to the seventh edition, that Dr. Underwood was the first to recommend the frequent use of calomel, both in large and frequent doses, in the treatment of the diseases of children. This very valuable medicine had not been advised by any other writer, before the appearance of the first edition of his work. That this favourite, and now too freely applied, remedy, has frequently been carelessly and indiscriminately resorted to, is very certain; but we are much indebted to Dr. Underwood for having first publicly stated the power which the judicious use of calomel possesses of alleviating, and even curing, many diseases of early life.

Some difference of opinion exists as to the advantages of administering Antimony to children. Dr. Underwood conceives that this medicine is too indiscriminately condemned in the "London Practice of Midwifery."

"The work here alluded to is "The London Practice of Midwifery," first published in 1803. It is a surreptitious copy of the late Dr. Clarke's Lectures, taken by a pupil, who committed very gross mistakes in various parts, and in others made the subject quite unintelligible. The merits of the work have notwithstanding carried it through five editions, in the latter of which many of the mistakes and errors have been corrected.—S. M." (P. xviii. *note.*)

Dr. Clarke makes the following remarks upon this subject:—"In the early stage of bowel complaints in infants, no remedy is so useful as an emetic; and ipecacuanha is the best and safest emetic that can be given. Tartarised antimony is sometimes dangerous, and there are instances recorded where children have died under its operation, even when given in very small quantities. In one case, two grains were dissolved in an ounce of water, of which half a tea-spoonful was given every quarter of an hour till it produced vomiting, which, when it took place, never ceased till the child died. Such violent effects never happen from ipecacuanha; two or three grains of which may be given every quarter of an hour till it vomits."

Dr. Merriman has "been induced to insert this extract, because his own experience of the occasional ill effects of antimonial preparations agrees with that here recorded. He has several times witnessed mischievous consequences from the indiscreet use of this remedy. The mode in which it is often directed to be given is a very dangerous one. "Let it be given every ten or fifteen minutes till it produces vomiting," is a very common, but very injudicious, prescription. No emetic ought to be persevered in if the child, after two or three suitable or usual doses, does not cast its stomach,—unless under the especial direction of some skilful practitioner. The indiscriminate use formerly of antimony, and now of calomel, has been, and still is, productive of much evil."

The practice of giving an emetic "in the early stage of the bowel complaints of children," is not generally adopted by the practitioners of the present day, although it is sanctioned by the authority of Dr. Clarke. With few exceptions, more benefit will be derived from the skilful management of purgatives. — no doubt.

Dr. Underwood offers many useful observations upon the plan to be adopted when infants are apparently "still-born." His success was great in such cases, and, in his own opinion, was owing to "unwearied assiduity and perseverance in his attempts, whenever there were no certain signs of death." The means to be employed on such occasions "consist only of warmth, clysters, stimulants, and especially blowing forcibly into the trachea." Dr. Merriman observes, "that a very general excitement of nervous energy is frequently produced by gently rubbing and irritating the soles of the feet with a nail or tooth-brush." It is not a little remarkable that, amongst the means of *rousing* apparently still-born children, Dr. Underwood should enumerate *tobacco* clysters. Dr. Merriman very properly condemns the practice, by observing, "that tobacco clysters so often produce syncope, that it seems extraordinary they should be recommended in such a case as this. All direct sedatives, of which tobacco is one, are likely to be prejudicial in this state."

Upon the subject of "bringing up children by hand," we have the following important statement from Dr. Merriman, which we submit to our readers, although it by no means accords with the result of our own observations.

"It has been a part of my duty (says Dr. M.) to endeavour to ascertain the amount of mortality among infants from this source; and, after much careful inquiry and investigation, I am convinced that the attempt to bring up children by hand proves fatal, in London, to at least seven out of eight of these miserable sufferers; and this happens whether the child has never taken the breast at all, or, having been suckled for three or four weeks only, is then weaned. In the country, the mortality among dry-nursed children is not quite so great as in London, but it is abundantly greater than is generally imagined. The summer is the most favourable season for making the attempt; but, if parents were fully aware of the hazard to which their children are exposed in the endeavour thus to bring them up, they would rarely choose to place them under the care of the dry nurse." (P. 3.)

We are fully sensible of the moral obligation which is imposed upon every mother of suckling her own child, if possible; but circumstances may occasionally render it necessary to bring children up by hand, and, provided the food administered to them be proper in quantity and quality, we really should not be inclined to rate the average loss so high as that stated by Dr. Merriman.

Dr. Underwood observes, that "it has long been suspected, and of late years more generally imagined, that some of the worst fevers, and more rare ill effects of child-bearing, may be prevented by suffering the milk to flow duly to the breasts, and be freely drawn from them, though only for the month. These advantages, one should hope, might tend to induce ladies of rank to set a general example, by performing this kindest and most pleasant office, at least during the month." Upon this point Dr. Merriman observes—

"I cannot agree in opinion with Dr. Underwood, that it is right to recommend ladies to suckle, 'though only for the month,' if they do not intend, or have already determined not to persevere in performing that office. On the contrary, I think, if they are to suckle their children 'only during the month,' that it is better, both for themselves and their infants, (who under these circumstances are never half suckled,) not to make the attempt for that short time.

"The first month of suckling is confessedly attended with more of uneasiness and suffering than is afterwards felt: indeed, after the first difficulties are overcome, this duty is accompanied with sensations of the greatest pleasure and delight; and it seems cruel to require of the mother to undergo all the trouble and pain of suckling, when she is to be debarred of all its pleasures.

"But we are told, 'it has long been suspected, and of late years more generally imagined, that some of the worst fevers, and more rare ill effects of child-bearing, may be prevented by suffering the milk to flow duly to the breasts, and be freely drawn from them, though only for a month.' If this be only suspected and imagined, much dependence cannot be placed upon the opinion. As regards the mammary abscess, I have much more frequently witnessed its occurrence when the mother has attempted to suckle for the month only, than when she has altogether declined putting her child to the breast. Certainly, if the mother is not to nurse her babe, particular care ought to be taken to guard against fevers, and other ill consequences; and, with such care, they are not much to be dreaded. The suckling of an infant is too serious a matter to be played with; and, if there be not a reasonable hope that the mother will be able to perform this duty completely, she had better at once resign the charge." (P. 5.)

Dr. Underwood was a zealous advocate for the use of the cold bath in the general management of children; and it is only to be apprehended, in the opinion of Dr. Merriman, "that on many occasions the rule he has laid down has been remembered, while the very important exceptions have been overlooked or forgotten, and the consequence has been too often the abuse of a beneficial practice." We perfectly agree with Dr. Merriman upon this subject. The popular and injudicious use of the cold bath, and exposure to cold air, with the view of "bracing" the child, whatever may be its constitution, are prejudices which have taken too firm a hold of the public to be willingly abandoned; and Dr. Merriman is doubtless correct in supposing that the "strongly expressed opinion of Dr. Underwood, of the absolute necessity of inuring very young infants to endure the cold air, as essential to their health, has been productive of great and extensive mischief."

Dr. Merriman offers some remarks upon the subject of abstracting blood from children, which are too practically valuable to be passed over.

"As it is always difficult, and sometimes impossible, in very young subjects, to draw blood by opening a vein with a lancet, it is usual to employ either leeches or cupping glasses for this purpose.

"Leeches are commonly preferred, being thought a less severe remedy than cupping, though in fact they frequently prove more severe and troublesome. The chief objections to the use of leeches are—1st. The difficulty of applying them. 2dly. The length of time which is consumed while they are drawing, and afterwards while a sufficient quantity of blood is flowing from the orifices; a length of time during which the active employment of other indispensable remedies is prevented. 3dly. The great uncertainty as to the quantity of blood obtained: this being sometimes so inconsiderable, as not at all to answer the purpose; at other times so great and uncontrolable, as to exhaust and debilitate the patient excessively. Instances are not rare in which, from the neglect of putting a timely stop to the flow of blood, infants have been actually destroyed by the gradual but overwhelming loss.

"Whenever a good cupper is to be met with, this mode of procuring blood is greatly to be preferred. He can apply his cups upon any part that may be required, and will draw blood from infants, even during the month, with great address and expedition. He will take away the exact quantity prescribed, even to a quarter of an ounce. The operation is quickly over, and, of course, the advantage of taking away the necessary quantity at once is obtained. There is no delay in employing other appropriate remedies; nor is there the fear that, through the practitioner's absence, the child will sink under the profuse discharge of blood, which has sometimes oozed, unobserved or unattended to, from the bites of leeches.

"In determining the quantity of blood to be taken away, so much depends upon the peculiar case to be treated, as well as upon the constitutional and relative strength of the child, that no precise rule can possibly be laid down. The attending practitioner must exercise his best judgment in directing the proper quantity, and he will often find it expedient personally to superintend the operation of cupping, in order to insure the complete effect which he expects from the loss of blood. It may, however, be useful to remark, that, during the first six weeks of life, from five drachms to an ounce of blood will commonly relieve the inflammatory symptoms; from six weeks to three or four months, one ounce, or an ounce and a half, will answer the purpose; and in this proportion bleedings may be adopted at subsequent periods of infantile life." (P. 299.)

Amongst other remedies for Scald-head, Dr. Underwood mentions the external application of a strong decoction of tobacco. Dr. Merriman observes, that a case of stupor ending in death occurred a few years ago at Shoreditch, from the incautious manner in which a father washed the head of his child with that application.

Ten or twelve cases of Imperforate Rectum have occurred in the practice of Dr. Merriman.

"In several of these cases, the delay of the operation appeared to be the principal cause of its failure. But I attribute the failure more frequently to the mode of operating, which was generally by passing a trocar, sometimes of too small a size, into the rectum: this never failed to give a temporary relief to the symptoms, by producing an evacuation of the meconium, but the aperture could seldom be kept properly open, though bougies and sponge-tents were used to preserve a passage. In one instance of this kind, the child lived six months, and then died, with an immensely distended abdomen.

"In two cases a perfect cure was effected: these children were both operated upon by the late Mr. Chevalier, who, with a scalpel, made a very free incision through the integuments, till he distinctly felt the fluctuation of meconium in the rectum; he then carried his instrument through the membranous expansion, or pouch, in which in these cases the rectum generally terminates; taking care effectually to divide it by a crucial incision, thus completely destroying its valvular structure. Both these children recovered without difficulty, and possessed the faculty of retaining, or expelling, the fæces nearly as well as if no malformation had ever existed. One of them is now fifteen years of age." (P. 616.)

We have presumed that a detailed review of Dr. Underwood's work could not be expected of us. We have, therefore, touched only upon those few points upon which Dr. Merriman has very briefly stated his opinion. In many respects the present edition has been much improved. The work was formerly printed in three duodecimo volumes, it is now thrown into one octavo. Dr. Merriman has also very properly given the "directions for the management of infants from the birth" at the commencement of the book. The consideration of such a subject ought certainly to precede, rather than to follow, a Treatise on the Diseases of Infants, although, in Dr. Underwood's editions, it occupied the last volume.

Notwithstanding Dr. Merriman has pruned the style of Dr. Underwood, by cutting out a number of such expressions as the "*little* smiles, *little* tears, *little* bowels," &c. &c. of the infant, the composition of the work is still trifling and faulty. If Dr. Merriman had favoured the profession with an original Treatise upon the Diseases of Children, it might probably have been of less use to "discreet and intelligent mothers of families," because the subjects would have been treated in a more scientific manner, and the performance would doubtless have been above the level of female readers, but it would have been a much more valuable addition to the professional library than a revised and improved edition of Dr. Underwood.

The blank in English medical literature, which has long been regretted, is yet to be filled up; for we cannot, in justice to our readers, recommend the volume before us as a full and complete treatise on the diseases of children; although we willingly admit that it contains some useful information, and that Dr. Merriman has much improved upon the former editions, both by his additions and omissions. In our opinion, however, he has been much too sparing of both.

*A Nosological Practice of Physic, embracing Physiology.* By GEORGE PEARSON DAWSON, M.D.—8vo. pp. 380. Longman and Co. London.

THE present work, we are informed in the advertisement prefixed to it, is the result of twenty-eight years dedicated to the study and practice of medicine. The object of the author is to exhibit general views of the principles and treatment of diseases, interspersed with classical, physiological, and pathological facts and observations. "Dissatisfied with existing Nosologies, he has constructed one for himself, which is simple, intelligible, and consentaneous with his own experience."

In the arrangement adopted by Dr. DAWSON, five orders are admitted: 1st. Febrile Diseases; 2d. Inflammatory Diseases; 3d. Nervous Diseases; 4th. Cachectic Diseases; 5th. Functional Diseases. The whole of the species and varieties of each order are arranged under a single genus. The generic definition is first briefly stated, and the species and varieties are then separately considered. It is obviously necessary that some arrangement of diseases should be adopted, particularly when we undertake to impart to others the information we may have derived from experience, or the result of our own reflections. But, to speak sincerely, we are not very anxious upon the subject of the mere arrangement of diseases. The simpler the classification, the better. Nosology can but embrace the boldest forms of disease. There must be varieties of every malady constantly occurring, which no nosologist can anticipate; and, consequently, all the attempts that have been made to enumerate every possible variation of disease have been unsuccessful, and have only served to increase the already overloaded vocabulary with words that many never understand, and few ever remember. The great end of all our labour must be the cure of disease; and how often, we would inquire, do we find ourselves assisted at the bedside of the patient by artificial arrangements? They may, to a certain extent, simplify the labours of the student; and this is the only use we can fairly assign to them.

Dr. Dawson's division is simple and perspicuous, and is well adapted for a work intended to give only general views of disease. Like other works on the Practice of Physic, the volume commences with the subject of Fever. Dr. Dawson takes but a very superficial survey of it. He does not even touch upon many important questions relating to it, which, although they have excited the attention of numerous writers, are still left in doubt, and would consequently admit of still further investigation. The following passage contains an opinion in direct opposition to most authorities, who have particularly treated of the various functional or structural derangements so frequently consequent to remittent fevers.

"When marsh fevers have affected the system for a very long time, they too often induce considerable debility, hepatic obstruction, visceral disease, and dropsy; or they are combined with pulmonic affections, obstinate dysenteries, or incontrollable diarrhœas, some of which are usually beyond the ordinary control of medicine. I never knew mercury productive of the smallest benefit: it only added weakness to weakness, and accelerated, rather than retarded, a fatal termination; yet calomel to affect the bowels, opium to remove pain or assuage distress, and warm port wine to impart comfort, have, in my own practice, certainly not limited, succeeded beyond my hopes." (P. 17.)

In our opinion, the exhibition of small doses of mercury, together with other appropriate treatment, is very necessary in most cases of local disease, or derangement of function, induced by intermittent or remittent fevers.

We must also enter our protest against the treatment recommended in that species of fever denominated Synochus. "The treatment of synochus consists in an emetic, if there be nausea or uneasiness in the stomach; *a large bleeding at the onset of the febrile attack*, and that repeated according to circumstances; together with liberal purging, and blisters for local pain." Possibly, the use of the lancet at the commencement of simple continued fever may be sometimes necessary; but it must be in cases where the general symptoms indicate synocha rather than synochus, or where some particular organ is evidently labouring under inflammatory action. If the general rule laid down by Dr. Dawson were acted upon, and "a large bleeding" abstracted at the commencement of every case of simple continued fever, we are convinced much mischief would ensue. We confess our observation is founded upon the inferences we have drawn from watching the progress, and treatment required, of the continued fevers of this crowded metropolis. Dr. Dawson's advice, however, is delivered too exclusively: no exceptions whatever are made to the treatment he recommends.

We agree with him in the propriety of administering emetics at the beginning of fever, if there be symptoms of gastric derangement. Fashion, which bears as much sway in medicine as in manners, has, in the present day, almost driven emetics from our list of remedies in fevers. In many cases of simple continued fever, the active interference of the physician will not be required. He should at all times watch with attention the progress of the symptoms from day to day; but when nature herself is evidently, although gradually, operating a restoration to health, he should deliberate maturely before he opposes her steps, by adopting what is termed a decided mode of practice, which may, it is very true, sometimes give a momentary eclat to the character of the practitioner, but more frequently proves prejudicial to the patient.

We observe Dr. Dawson pays Dr. CLUTTERBUCK a very curious kind of compliment. "Dr. Clutterbuck, with an ingenuity and talent which do him honour, has asserted and attempted to prove fever to be, at all times, an inflammation of the brain.—This will not do: it is to mistake an occasional effect for a constant cause. But for this unhappy prepossession, he would have ranked high among the authors on Fever; since it is not easy to discover his superior." If Dr. Dawson were to employ a builder to erect him a substantial dwelling, the foundation of which was afterwards discovered to be unstable, would he complacently remark, "he would have ranked high among the builders of houses, but for the single fault of laying a rotten foundation?" The cases appear to us to be parallel: both the house and the hypothesis would be remarkably good, if they had but a foundation.

Rubeola is said by the author to occur only once during life. It is, however, of much importance for practitioners to remember that several exceptions have occurred to this general rule. Dr. BAILLIE has published some cases of secondary measles; and, in our own more limited experience, we have seen several similar instances which were too strongly marked to admit of doubt. The black measles, mentioned by WILLAN, and of which no case has fallen under Dr. Dawson's notice, is by no means common. Whenever the eruption does assume a darkened hue, much apprehension is entertained by the friends of the child. There is, however, no greater degree of danger in these cases than in the usual forms of the disease. We are told that "blood-letting may be safely and expeditiously performed in the veins of the hand" in children. We confess we are very sceptical upon this point.

Dr. Dawson not unfrequently amuses us by the brevity and ease with which he dismisses many questions, that other writers have considered involved in much difficulty. For example, after having given twenty lines to the consideration of Variola, without a word upon the modified disease which occurs after vaccination, he observes, that "the symptoms narrated will sufficiently distinguish varicella from variola."

If he had entered upon the task of distinguishing from varicella the modified variola to which we have referred, he would perhaps have confessed that the diagnosis is not so easy.

Upon the subject of Otitis we find some pertinent remarks.

"Inflammation of the ear, although an apparently unimportant affection, is always to be narrowly watched, since, from its proximity to the brain, fatal consequences may arise. Two cases of this nature terminated in death, under the care of my friend Dr. Brown, of Sunderland, whose acuteness and talents render him an ornament to the medical profession. The first was a young man, who was affected with purulent discharge from, and excrescences in, each ear. In London he used strong injections, composed of the sulphate of copper, which considerably lessened the discharge, and he returned under the impression of having been cured. Soon after, he was seized with phrenitis, to which he fell a victim. A middle-aged lady was the subject of the second case: in her, otitis advanced into phrenitis, and destroyed life in a few days.—In both cases the most active treatment was pursued: liberal venesection produced striking, but only temporary, relief.

"In otitis, the ear should be fomented, or warm water syringed into it; the bowels freely opened; and leeches, as well as a blister, applied. But, whenever the face is flushed, the pulse quickened, and the pain severe and shooting into the head, liberal venesection is demanded, and ought to be repeated, if the urgency of the symptoms be not checked; for, in some instances, universal pain, delirium, and coma supervene. Where suppuration threatens, warm poultices must be applied, and likewise tepid injections used. Occasionally, suppuration proceeds to the complete destruction of the whole of the internal ear, and the bones are discharged through the meatus auditorius, with much purulent and fetid matter. Here little is to be done beyond attending to the general health, and the adoption of strong injections of oak-bark and other astringents." (P. 60.)

Dr. Dawson is of opinion, "that, when phrenitis appears, it is impossible to ascertain whether the membranes or the brain itself are inflamed: nor is it of any importance, since the treatment does not vary. The probability is, the membranes are first inflamed, and that the inflammation, if not speedily subdued, extends itself to the substance of the brain." We believe he is correct upon this point, notwithstanding the attempts that have been made by many of the French pathologists to distinguish the particular seat of inflammation, and the numerous divisions they have proposed, each designated by its corresponding appellation. Can any thing but ignorance or affectation induce any man to pretend that he can, during life, distinguish inflammation of the arachnoid from that of the pia mater?

After having given a slight sketch of the progress and treatment of Enteritis, Dr. Dawson introduces the following case, which should be attentively considered, particularly by junior practitioners, whose reputations would materially suffer from the loss of a patient under such circumstances. Upon this very important subject we may refer our readers to the interesting little volume of Dr. MARSHALL HALL, which we noticed in our Number for December. The case related by the author shows that not only life, but even rationality and locomotion, can go on under enteritic gangrene.

"Mr. N—, aged thirty-seven, in December, 1818, after having been very cold the day before, was seized with enteritis: the usual practice was employed, and in five days he was pronounced convalescent. He remained weak, free from pain, speaking in a whisper, from Thursday to Sunday; and on that day he dined in another room with his wife and friends, ate the wing of a chicken, and drank a glass of wine. At one on the following morning, when all was sunshine, he was almost instantaneously attacked with agonizing pain in his bowels, and insuppressible inclination to go to the night-chair. The pain increased, he sank rapidly, was covered with clammy sweats, and died in five hours! On dissection, the colon was found black in several places; and there were a few holes near the sigmoid flexion, through which a quantity of hardened feces had passed into the pelvic cavity, and were floating in fluid. The cavities of the thorax were full of water.—In this unhappy case, in consequence of the cessation of pain, gangrene had been mistaken for convalescence; and it is obvious that the agonizing pain, prior to dissolution, arose from the separation of the dead from the living intestine." (P. 91.)

"Pancreatitis," says Dr. Dawson, "is a stranger, introduced by myself into the temple of Nosology, where it has an undoubted right of admission, although unjustly excluded by several nosologists." We find, however, that inflammation of the pancreas is also mentioned by Dr. GOOD. The disease is very rare, and perhaps never indicated by any symptoms which could lead us to detect its existence. "The temple of Nosology," therefore, is not much honoured by the introduction of such "a stranger."

To obviate the distressing pain that in general accompanies Nephritis, we are perfectly aware of the necessity for the free exhibition of opium, after the treatment required for other inflammatory affections, with the exception of blisters; but we apprehend our author is imprudent in recommending "not less than two drachms of the tincture of opium every two hours, until a sensible effect is produced." In many cases, we should find the first *sensible* effect would be the *insensibility* of the patient, notwithstanding the modifying influence that pain certainly exerts over the operation of narcotic medicines. We have seen many cases of nephritis, and have succeeded in relieving the sufferings of the patients by much smaller doses. We should not prescribe more than thirty or forty minims as a first dose, and afterwards, if necessary, ten or fifteen minims every two or three hours until relief is obtained. Occasional exceptions may doubtless arise, in which a more liberal use of opium may be demanded.

While upon this subject, we may throw in an observation that may not be considered altogether irrelevant, with respect to the relative power which the Tincture of Opium and Battley's Liquor Opii Sedativus possesses in relieving pain. For this purpose, the latter preparation is decidedly inferior to the former, even in larger doses, although it has the advantage of not producing constipation of the bowels, nor that heaviness of head and clammy state of the mouth, which are well known to arise from laudanum. In many cases, when we have no severe pain to conquer, it is a nice question to determine whether any preparation of opium can with propriety be employed for the purpose of diminishing excessive irritability; and in such instances we should certainly prefer the preparation of Battley, particularly if the action of the heart and general vascular system appeared to be increased.

Passing over several very superficial sketches of other inflammatory affections, we arrive at "Order III. Nervous Diseases," the "generic definition" of which is—

"The brain, nerves, and muscles, singly or simultaneously disordered; denoted by aberration of mind; loss of sense, of voluntary motion, and stertorous breathing; depravation or abolition of the sense of external parts; affections of the nerves or muscles." (P. 177.)

Upon the subject of Mania, Dr. Dawson opposes himself to the peculiar views of Mr. LAWRENCE and other authorities, who contend that madness is the effect of disease of the brain. The dogmatism with which Dr. D. delivers his opinions is much to be lamented. It would not require the acumen of Mr. Lawrence to point out the many inaccuracies with which his observations upon mania abound. We cannot enter at large upon so extensive a question, but we may inform Dr. Dawson that he is in error when he states that "every enlightened man in the medical profession" will deny that "disease of the brain, or its membranes, is commonly discovered;" and he is, *a fortiori*, wrong in asserting that "it is admitted, most unequivocally, that disease of the brain is rarely met with in maniacal patients." The question is still involved in doubt, but there are as many authorities who contend that some disease or preternatural condition of the brain is the usual cause of mania, as there are in support of the dogma of Dr. Dawson. As a proof that "every enlightened man" does not acquiesce in his views, we shall quote but one writer, although it would be easy to accumulate evidence upon this point. HASLAM,* after having related many cases, observes, "From the preceding dissections of insane persons, it may be inferred that madness has always been connected with disease of the brain and of its membranes. Having no particular theory to build up, they have been related purely for the advancement of science and of truth. It may be a matter affording much diversity of opinion, whether these morbid appearances of the brain be the cause or the effect of madness. It may be observed, that they have been found in all states of the disease. When the brain has been injured from external violence, its functions have been generally impaired, and inflammation of its substance, or more delicate membranes, has ensued. The same appearances have, for the most part, been detected when patients have died of phrenitis, or in the delirium of fever: in these instances, the derangement of the intellectual functions appears evidently to have been caused by the inflammation. If in mania the same appearances be found, there will be no necessity of calling in the aid of other causes to account for the effect: indeed, it would be difficult to discover them."

Dr. Haslam then proceeds to argue the point with those who suppose *a disease of the mind,* but with a deference for the opinion of others, which Dr. Dawson would imitate with advantage. "When we find insanity," says Dr. Haslam, "as far as has been hitherto observed, uniformly accompanied with disease of the brain, is it not more just to conclude that such organic affection has produced this incorrect association of ideas, than that a being, which is immaterial, incorruptible, and immortal, should be subject to the gross and subordinate changes which matter necessarily undergoes."

Continuing his observations upon the subject of mania, Dr. Dawson, with the same authoritative tone, "meets Mr. Lawrence on his own ground, and tells him that *none* but mental remedies are of *any* use in *every* species of mental derangement." We know not the extent of the author's experience, and cannot but regret that the sources from

* Observations on Madness, 2d edit. p. 238.

whence his opinion is derived are not stated. Highly as we estimate the importance and necessity of moral treatment in every kind of mental aberration, we have yet to learn that its application, however judiciously directed, is to be trusted to *alone,* and *unaided* by other auxiliary remedies.

In the section on Convulsion, the subject of puerperal convulsions is incidentally noticed, and again we have to lament the decisive tone with which opinions are delivered upon a subject of such vast importance. After having had recourse to blood-letting, which is to be promptly and largely employed, "if delivery be not effected, or the placenta not extracted, manual efforts must be adopted." It is probable that Dr. Dawson's book may not be particularly consulted as an authority upon this point, and we would guard the junior members of the profession not hastily to act upon the crude direction of interfering by "manual efforts," which may or may not be required; nor, without very mature deliberation, "to ask for a pair of scissors, thrust their points into the head of the child, and scoop out the brain with their fingers," when the head of the child is "without the mouth of the uterus." We have had considerable experience in the practice of midwifery, but it has never yet fallen to our lot to be obliged to have recourse to the horrible duty of destroying the child in cases of puerperal convulsions. We know that such cases may occur; but we must be allowed to reprobate, most forcibly, the manner in which the case is related in which Dr. Dawson, with so much promptitude, "scooped out the brains with his fingers." Such a case ought to have been very circumstantially detailed, and might, with much propriety, have been accompanied by some observations from a man of Dr. Dawson's age and experience, for the instruction of his junior brethren, that they might not rashly or unjustifiably have recourse to so responsible a proceeding.

"Order IV. Cachectic Diseases."—Phthisis Pulmonalis. We are inclined to agree with Dr. Dawson in his restriction of this term. The loose and indefinite manner in which it is often applied by many practitioners, not unfrequently leads them to form an erroneous estimate of the value of particular remedies, and of their own successful practice in this formidable disease.

"The amiable and venerable Dr. Duncan, of Edinburgh, has divided phthisis pulmonalis into three kinds,—namely, the *catarrhal, apostematous,* and *tubercular;* and, agreeably to his views of the subject, he might have added a fourth, and designated it the *hemoptyst.* The hemoptyst arises in this manner:—A person, most generally of a bad or weak constitution, suffers an attack or attacks of hæmoptysis; but a vessel, or perhaps vessels, in his lungs, instead of healing, degenerate into a small ulcer or ulcers; and thus originate cough, expectoration, irritation, and other symptoms. It was the frequency of this occurrence which led Cullen to contemplate phthisis as nothing more than a sequel of hæmoptysis. For my own part, I attach no importance to these divisions, and consider them, with the exception of the tubercular, to be fundamentally erroneous. Let us pursue this subject farther, to prevent misconception, and to establish precision. There is only one kind of phthisis pulmonalis, and that is the tubercular, which may be conveniently and practically divided into two stages: the first, the occasional formation and inflammation of tubercles in the lungs; the second, the suppuration of them. The tubercles in a suppurative state constitute genuine pulmonary consumption: there is no other." (P. 272.)

We observe nothing that particularly requires our attention in the consideration of "Order V. Functional Diseases." Upon the subject of Sterilitas, the following remarks are made, which are probably not without foundation.

"An honest man is seldom a father in the year he is made a bankrupt. Severe intellectual exertions on the part of the husband will frequently cause temporary sterility in the wife, which will continue until such exertions have ceased. Dr. G——, now of London, who at this moment is considered by many as one of the ornaments of the medical profession, was married, in 1811, to an interesting and lovely young woman, by whom he had two fine children. He commenced author; bestowed great intellectual labour on three successive works, all well known, and one abundantly praised by the medical world; and, when he took up his pen, his wife became sterile, remained so from 1814 to 1818, and then became pregnant, after the publication of his last work.—Again, the fruitful wife of an half-pay officer or distressed gentleman becomes suddenly barren, and continues so; but no sooner does hope show itself, and prospects brighten, than pregnancy takes place, even to the amount of twins. These facts have occurred under my own observation." (P. 374.)

We would not conclude that a man may, to a certainty, stop the too-rapid increase of his domestic circle by turning author. If such were the fact, the press would groan with the labours of men, who, from the *res angustæ domi,* were induced to contemplate a rising family with anxiety and alarm.

We scarcely know for what class of readers Dr. Dawson's volume is particularly adapted. It is a mere text-book. Almost every subject is dismissed with the "Spartan brevity" which he mentions in his discussion of inflammation of the pleura. He who wishes to meet with a very slight view of the "head and front" of diseases, will be gratified by the perusal of it. As a "Practice of Physic," it appears to us decidedly inferior to others with which our readers are well acquainted. We have more than once had occasion to notice the magisterial and unyielding tone in which Dr. Dawson conveys his opinions: this is the prominent fault of the work. The descriptions of the nature and treatment of disease are, with some exceptions, as correct as they possibly could be, when treated in so superficial a manner. Dr. Dawson has but glided along the surface, either in the enumeration of facts or the discussion of theories.

---

*An Oration, delivered on Thursday, February* 9, 1826, *before the Hunterian Society: with Supplementary Observations, and Engravings.* By Sir William Blizard, Knt. F.R.S.; F.A.S.; F.R.S. ED.; Soc. R. Gotting. Corresp.; Hon. Prof. of the Royal College of Surgeons in London; Surgeon to his Royal Highness the Duke of Gloucester, and to the London Hospital.—4to. pp. 44. T. and G. Underwood, London.

We look upon the productions of the veterans in our profession as always entitled to our respectful notice. With regard to the "Oration" before us, we are not disposed to enter into any critical analysis of its merits, but shall extract one or two passages, which appear to us of sufficient interest to be laid before our readers.

Physiological errors, it is observed, lead to dangerous consequences, and the following illustrations are given:

"Crimson lines, in absorbent vessels leading to lymphatic glands, are commonly supposed to indicate absorbed virulent matter: whereas, the speaker, from long and steady observation, can aver that the contrary is the general truth.

"If there be, in any mind, doubt on this point, how important that it should be removed; and that opinion in the particular case should rest on universal admission of fact!

"How often have the lives of men, beloved and revered for their virtues and scientific endowment, fallen sacrifices to different, yet equally erroneous hypotheses, relating to expressions of actual, or apprehended, virulence by absorption?

"Let not the speaker be supposed to deny that expression of inflammation of an absorbent vessel may co-exist with the current of a poison in it: rarely, however, does such an expression occur, from the certain absorption of variolous matter, of syphilitic virus, or of the vaccine lymph; but frequently is the sign expressed from a wound by a clean metallic instrument, or from a fragment of undiseased bone.

"What notions are generally entertained of organic alteration of the prostate gland: how frequent the declaration of its schirrous condition! Not any part of the human body may have absolute exemption, by organisation and implanted disposition, from such a distinctive disease. But a long period of anatomical research affords to the recollection of the speaker only one instance of morbid affection of the prostate, which he could properly designate schirrus. The morbid character of schirrus does not admit of favourable alteration; but only to the carcinomatous condition. Other distinctions of induration, and enlargement, of the prostate gland, are within the sphere of curative consideration. How encouraging are such reflections to the persevering exercise of judgment and skill! How consolatory to sufferers, otherwise unsustained by the hope of relief?

"That revered promoter of anatomical and chirurgical knowledge, Cheselden, has somewhere recorded that the divided portions of a fractured patella would not become united by an osseous medium: and this opinion was maintained, at no great distance of time, by the excellent Mr. Warner.

"But a fractured patella will become united by a firm bony production, under the laws of ossification, which direct the process of union of every other divided bone. In every case of fractured bone, union will necessarily have relation; to the sphere of ossifying disposition of the preparative vessels, which in the patella appears to be very limited; to the proximity of the divided portions; and to various other relative circumstances; not to mention chirurgical treatment, as conformably or not to correct notions of muscular action.

"The physiological error, thus gravely sanctioned, would not be important as to the event of union of a fractured patella, whether by a bony or a ligamentous medium, as either would be efficient; but arguments might be maintained, and erroneous conclusions drawn, relating to the general ossifying power of the vessels of the divided parts of bone, from the admission of the error.

"The illustrious Pott has observed, that ligatures on the omentum proved fatal.

"If, in consequence of the excision of omentum in the operation for hernia, a considerable portion of omentum be included in a ligature, the effect upon the colon, stomach, and diaphragm, would be such as fatally to maintain, or to renew, the hernial symptoms.

"Whereas, should ligatures be made on single or detached vessels, the cords be loosely brought out, and the omentum returned in such a manner as freely to expand and float in the abdomen, no evil from the ligatures would ensue." (P. 14.)

With regard to Hydrophobia, Sir William states—

"The speaker, having had many lamentable opportunities of observation on hydrophobia, will attempt to cast a ray of elucidation on the subject.

"The first of the instances of hydrophobia which happened under his notice, long ago, was in a child, who had been bitten in the lip by a rabid dog. The wound healed kindly in a few days, by simple applications; and musk and cinnabar were internally administered by the attendant practitioner.

"The child was too young to have any consciousness of danger, nor had the parents apprehension of any, subsequently to the healing of the wound; until about the twenty-first day, when, the child having past two or three restless nights, the disease became decidedly formed, and soon terminated fatally.

"Assurances have been received of several occurrences of hydrophobia, from bites of rabid animals, in children too young to have been seriously impressed by the influence of the mind, and only sensible of painful feeling in the part affected. Such histories are against the admission, that hydrophobia is the consequence of workings of imagination.

"A young woman, servant to a clergyman at Hoxton, a patient of the late laborious cultivator of natural knowledge, Mr. Parkinson, had been in the practice of feeding and caressing a favourite dog. The animal became, and died, rabid, without her apprehension of its condition. The season was winter, and her hands were much chapped from cold. She had suffered the dog freely to lick her hands; but the creature had not bitten, nor had ever attempted to bite her. Nothing extraordinary occurred in her hands; but, after about three weeks, she was seized with unequivocal symptoms of hydrophobia, and thereupon sent to the London Hospital; where, after ineffectual endeavours for her relief, she died, a victim to her undistinguishing kindness.

"This case surely expresses disease by absorption. There was no painful infliction of wound to justify a supposition that the disease was the consequence of the local impression of a stimulus, *sui generis,* operating upon the nervous system, on the encephalon, and ultimately, with fatal influence, on various organs of the body.

"Writers of unquestionable veracity have asserted that, about the period of the accession of hydrophobia, the wounded part, which had been some time healed and easy, became painful, and manifested signs of irritation.

"The admission of the fact of a local morbid action, preceding or accompanying the symptoms of general influence upon the system, must necessarily incline the mind to a most important conclusion, strengthened by considerations of the analogies of events in the experiments of Hunter, relating to effects on the insertion of variolous matter; and since by remarks in the practice of vaccination.

"On reasoning *a priori,* without practical evidence, excision of the part bitten by a rabid animal would naturally be considered as the process which should be adopted, in prevention of the dreaded evil; and which, at any time before the morbid action has begun in the wounded part, would be performed with probability of success. Positive evidence can be only on the side of inefficacy: but the mass of presumptive evidence of success by the practice of excision, is so considerable as nearly to approach positive weight of argument.

"Towards confirmation of a process thus founded, the speaker can assert that, in the numerous instances of his performance of it at the London Hospital, and on private calls, at various periods from the time of the bite, not a single consequence of hydrophobia has occurred.

"Some surgeons have doubted the efficacy of excision, from their knowledge of its failure. But no person would pronounce this practice as invariably preventive of disease, who has well considered the different circumstances under which excision may be performed,—the divers susceptibilities of the human frame,—the possible varieties in the depth and extent of the wound by the bite, —the not improbable diffusion, far beyond the wounded part, of influence from the saliva of the rabid animal,—and the chance of inattention or unskilfulness in the performance of the operation, as often is illustrated in vaccination." (P. 25.)

An Appendix is given, containing observations on the effects of a stretching force applied to muscle, and on the treatment of a transverse fracture of the patella, which were read to the Hunterian Society on a former occasion. The following is a description of the method of treatment adopted.

"Not any bandage whatever is applied; even little regard is had to position: in most cases, however, the patient is kept lying, with the leg extended.

"Some time since, mention was made to several members of this Society, that there were then two cases in the hospital, under the explained treatment. One case terminated in a firm bony union of the divided parts; the other by a short ligamentous medium. Both patients left the hospital with perfect freedom of motion of the knee-joint.

"Circular bandage, especially if applied during any degree of inflammatory action, is not unfrequently the cause of adhesion and rigidity of the tendinous and surrounding cellular parts, very unfavourable to the motions of the limb.

"A lady received a fracture of the patella, which was treated by bandages; and, after a ligamentous union, adhesions were found to have been formed, and to prevent the motions of the joint. Many months after, she had an accident which separated the united parts of the patella, and loosened the adhesions: she was now treated without any bandage; the parts united, and her surgeon informed the author, a few days ago, that the functions of the joint were restored.

"The violent effort of the extensor muscles to save the body from falling, often occasions a separation of the broken portion of bone to a considerable distance up the thigh; where it would remain, unless removed by external means. But such removal is not in the least degree difficult, after the flurry of the muscular fibres has ceased. Gentle applications of the hand will effectuate the purpose, to the ordinary extent of elongation of the muscles.

"The author's treatment of rupture of the tendon of the extensor muscles of the leg, of the tendo-achilles, and of fracture of the olecranon of the ulna, has long been according to the expressed principle of conduct.

"The learned and highly respected rector of a neighbouring parish, had lately the tendon of the extensor muscles of the leg completely lacerated, in the like manner in which a transverse fracture of the patella happens. Not any bandage was employed: the divided parts became perfectly united, and the use of the limb restored.

"A few months ago a man was admitted into the hospital, on account of the division of the tendo-achilles, by a cutting instrument. The author directed all the bandages which he found applied to be removed; the wound to be dressed superficially, and the patient to be kept at rest in bed. The wound readily healed, and the patient left the hospital with the perfect use of the foot.

"One observation will decisively express the propriety of every rational endeavour to avoid circular bandage in a fracture of the patella. Such description of bandage is known to have impeded union in fractures of the bones of the upper and lower extremities; and must therefore be of injurious tendency, at least, in fractures of the patella." (P. 37.)

---

*Observations on the Artificial Mineral Waters of* Dr. Struve, *of Dresden, prepared at Brighton. With Cases.* By W. King, Fellow of the Royal College of Physicians, London; late Fellow of St. Peter's College, Cambridge.—8vo. pp. 53. Highley, London, 1826.

Dr. Struve, having suffered from paralysis of the left thigh and leg, from the injudicious use of prussic acid, repaired to Marienbad, for the purpose of drinking the waters. This he did with the effect of restoring his health; and he was led by this circumstance, and by the favourable opportunity afforded him, to turn his attention to the chemical composition and medical effects of this and other mineral springs in Germany. In the prosecution of these investigations, he seems to have conjectured that it might probably be advantageous to others, and profitable to himself, if he could succeed in imitating these productions. This required much perseverance and no small skill, but the result seems to have been satisfactory; and one of the ends in view, at least, has been accomplished, as pump-rooms were speedily established at Dresden, Leipsic, Berlin, Warsaw, and Koeningsburg, and "many hundreds of invalids have resorted to these institutions, in preference to the natural springs." But it appears that it is not necessary to travel into Germany, as Dr. Struve "has introduced among us one of the greatest blessings which this country has known in the present day."

Such is the opinion which our author does not "hesitate to express" of the benefits resulting from the institution at Brighton for mineral waters, after having had an "opportunity of observing their effects for only one season." Dr. King is very zealous in the cause, and, if the said institution should not draw together "many hundreds of invalids," it will not be for lack of his commendation. He is decidedly of opinion, however, that they "will not supersede the ordinary labours of the profession," (which, by the by, would be very inconvenient for practitioners residing at watering places;) but, on the other hand, he observes that—

"They are alterative, and, *in a certain sense*, tonic, for they seem to affect the system in the same way as alteratives do, by a gently purifying influence; and they gradually improve the strength, though not after the direct manner of the ordinary tonic medicines. The tonic effect is evidently the consequence of the alterative; for it is always the later of the two, and does not take place till the various functions of different parts are obviously improved. The alterative power seems to penetrate every organ and texture of the body, and to excite and regulate every secretion; while the tonic power seems to be the result of the restored healthy action of the body, similar to what takes place in a perfectly healthy constitution. In a healthy person, physical or animal power is the result of the general health of the body. So these waters, by their alterative power, restore every part of the body to a healthy state, and the effects of this are animal strength and vigour.

"The first perceptible consequences of taking the waters are very slight: they gently promote perspiration, and the flow of urine. These are their mildest effects. Perhaps, a person perceives the action of the bowels to be more regular and free, without being inclined to attribute it entirely to the water; though, upon reflection, he becomes convinced in a few days that it is so. The appetite and spirits improve in the same imperceptible manner; the nights are more composed, and the sleep sounder and more refreshing; then an unusual determination to some part of the body seems to take place, which is described as if all the small vessels of the part were roused from a quiescent languid state, into one of activity: this is accompanied by a sense of fulness and stretching, as if the parts might even give way; and, when the head is thus affected, the sensation is oppressive and painful. These effects require watching: if they pass on favourably, the disease is gone, the constitution renovated, and restored to vigour, strength, health, and spirits." (P. 8.)

And a general summary of the "remarkable and important" changes which they produce is thus given:

"1. They improve the qualities of the blood.

"2. They alter and improve the structure of the whole body of every part, and of every organ.

"3. They improve the vital powers of each part and organ.

"4. They improve the functions of every part and organ.

"5. The result of all this is, in one word, Health,—the health of every part,—and the health, tone, and vigour of the whole system, both of body and mind. Of this let those speak who have witnessed the effects; let those speak who have tried them. The medical world of London will soon have an opportunity of deciding for themselves, in the result of those cases in which they may prescribe these waters." (P. 13.)

As they do all this, it would be in vain to say any thing against them. The following, however, we can scarcely regard as an example of improvement in the structure of the parts, as stated under the second head; and we rather question whether the subject of the case looked upon it in that light.

"A Prussian general was going through a course of the waters: he had formerly had his arm broken in action, which had been set, and was become perfectly strong. After drinking the waters some little time, he perceived that the fractured part of the limb became tender and loose. The absorbents had evidently taken away the bony deposit by which the union had been formed, owing to the active energies imparted to them by the waters." (P. 19.)

In the body of the pamphlet are contained some desultory remarks upon the operation of medicines, from which we suspect that our author is a disciple of Dr. HANNEMAN'S, as he seems to be enamoured of minute doses of medicine; and subjoined are the cases of Dr. Struve and twelve others, very loosely detailed; the majority consisting of hysterical women, who would probably have got well during a trip to the seaside, with any medicine,—or with no medicine; with bread pills,—or with the Carlsbad waters of Brighton.

